# Sonographic findings of mass-forming extramammary Paget’s disease in the scrotum

**DOI:** 10.1259/bjrcr.20190018

**Published:** 2019-04-29

**Authors:** Young Sup Shim, So Hyun Park, Seung Joon Choi, Su-Joa Ahn, Yu Mi Jeong, Tae Beom Kim, Juhyeon Jeong, Hee Joo Kim

**Affiliations:** 1 Department of Radiology, Gil Medical Center, Gachon University College of medicine, Incheon, South Korea; 2 Department of Urology, Gil Medical Center, Gachon University College of medicine, Incheon, South Korea; 3 Department of Dermatology, Gil Medical Center, Gachon University College of medicine, Incheon, South Korea

## Abstract

Extramammary Paget’s disease (EMPD) is a rare cutaneous malignancy involving the scrotum and may be confused with other scrotal malignancy. We describe the sonographic findings of an extremely rare case of mass-forming EMPD of the scrotal wall. Ultrasonography, which shows mild heterogeneous hyperechoic masses with a stalk connected to the dermis, can help predict the depth of vertical invasion of the lesion. The lesion extent should be precisely evaluated because the presence of dermal invasion of EMPD is the risk factor in distant metastasis and is known to result in a worse prognosis. Ultrasonography is a primary imaging modality to evaluate the extent and vertical invasion of EMPD. Surgical local wide excision is the treatment of choice for EMPD and histopathology confirmed the diagnosis.

## Introduction

Extramammary Paget’s disease (EMPD) is a rare cutaneous malignancy. The common sites of EMPD are those rich in apocrine glands, such as the perineum, vulva, axilla, scrotum, and penis.^1^ EMPD of the scrotum was first reported by Crocker in 1889.^2^ The clinical manifestations of EMPD are erythematous, eczematous, or erosive lesions; pink or red plaques with scaling; and papillomatous surface of the skin.^3^ EMPD is a dermatologic disease; hence, only few reports to date have documented its radiologic findings.^4–6^ Herein, we report the sonographic findings of a case of mass-forming EMPD of the scrotal wall.

## Case report

**Figure 1.**
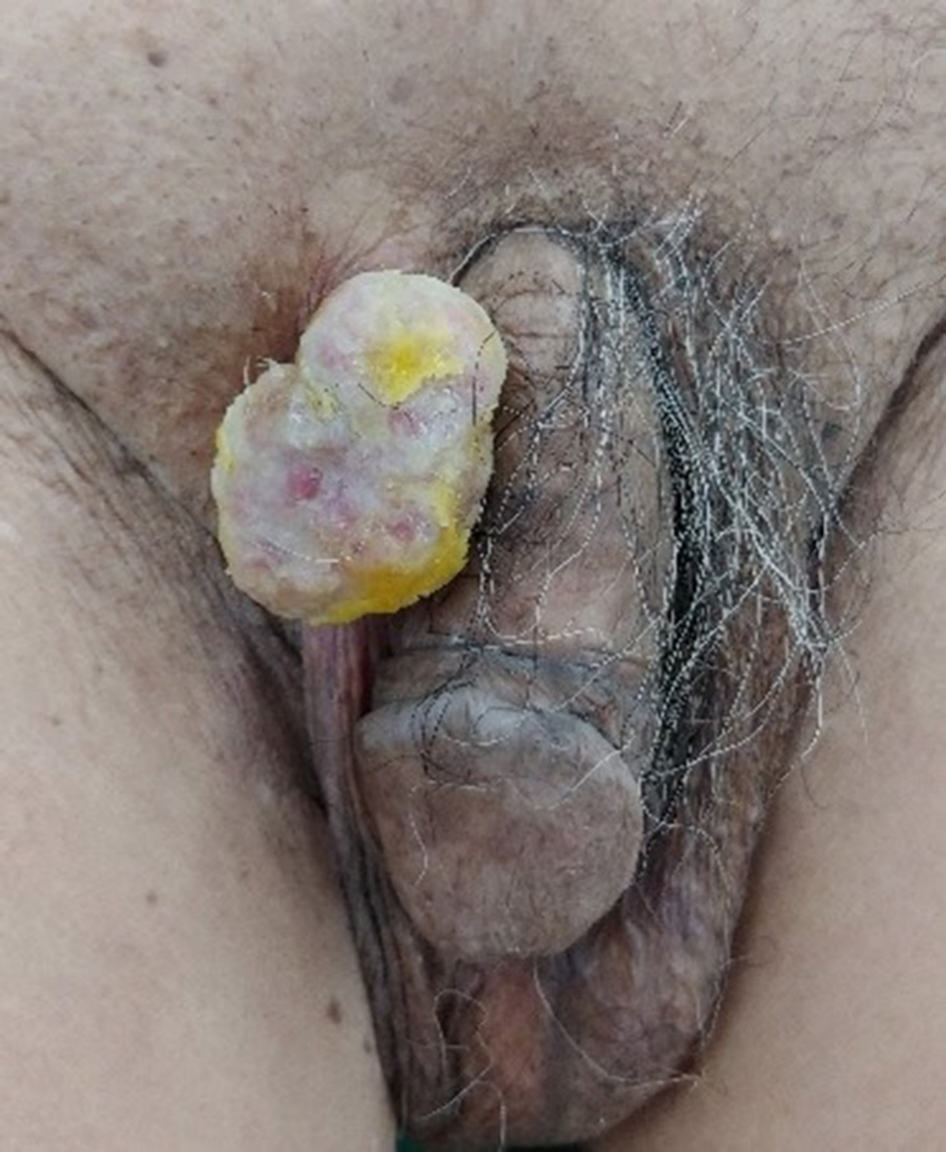
Photograph of the patient. A multilobular contoured whitish mass with small erosions in the right upper scrotum. No skin lesion is observed around the mass.

**Figure 2.**
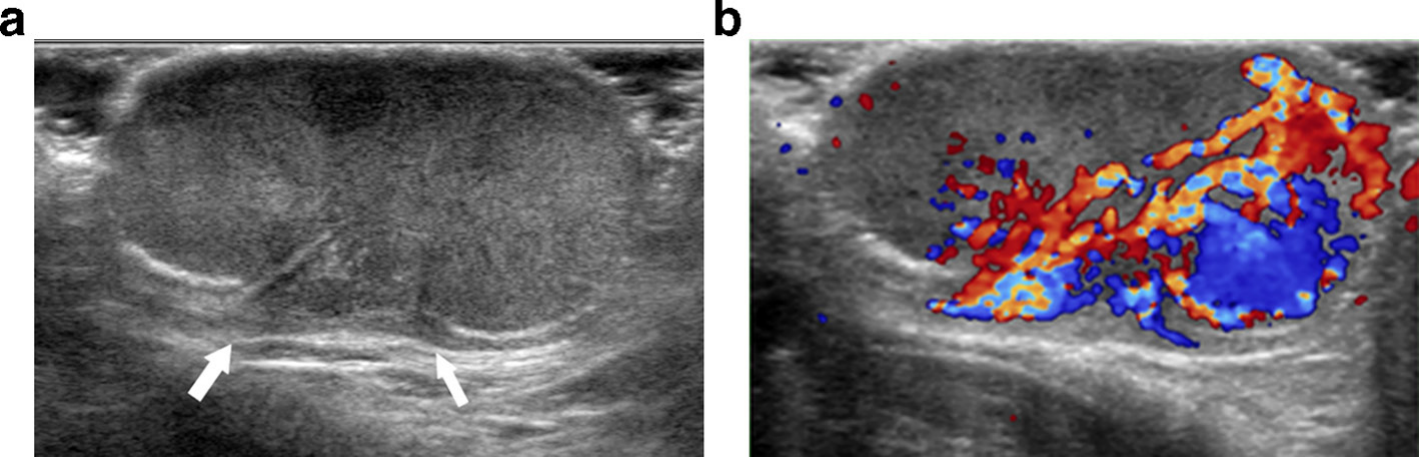
A, Ultrasonography shows a mild heterogeneous hyperechoic polypoid mass in the right upper scrotal wall. The stalk of the mass is connected to the dermis, but the subcutaneous fat is not invaded (white arrows). B, Color Doppler ultrasonography shows remarkable intratumoral vascularity in the peripheral portion of the mass from the stalk.

**Figure 3.**
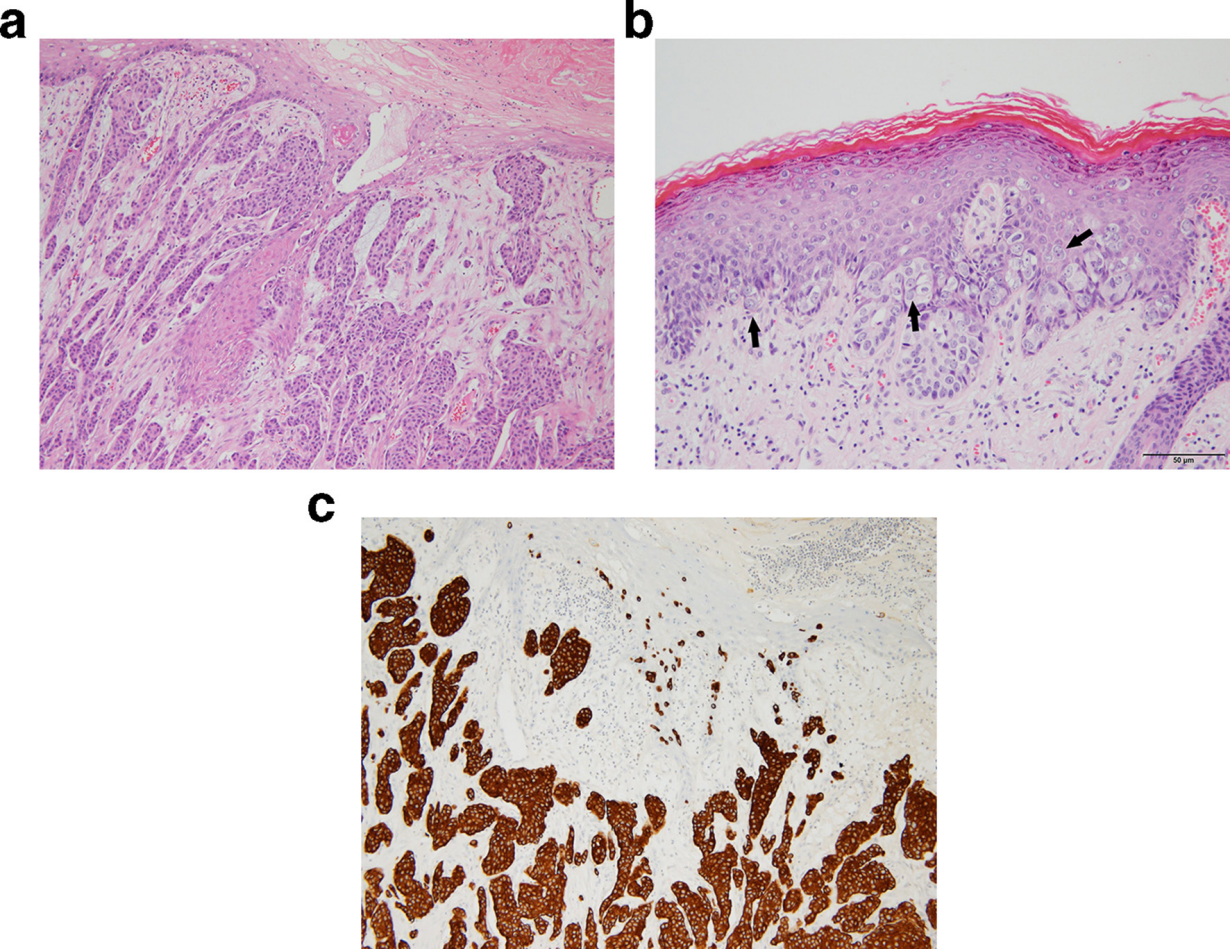
Extramammary Paget’s disease with an invasive component. A, Single and nested tumor cells are arranged within the epidermis and infiltrate into the dermis (hematoxylin and eosin staining). B, Large pale and vacuolated Paget cells are found mostly in basal layer of epidermis (black arrows). C, Tumor cells diffusely express cytokeratin 7.

An 84-year-old male presented to our hospital with skin erosion and a palpable mass on the scrotum. One year ago, the patient had undergone CO_2_ laser treatment for the mass at a local clinic, but recently, the size of the mass had increased. The patient had no symptoms such as pain, swelling, or itching associated with the mass. A visual evaluation revealed a multilobular contoured whitish mass with small erosions in the right upper scrotal wall (Figure 1). No additional skin lesions were observed around the scrotal mass. He had no other diseases, except Alzheimer’s disease. Moreover, no significant findings were found on complete blood count (CBC), chemistry profile, serum tumor marker tests (carcinoembryonic antigen [CEA], prostate-specific antigen [PSA], and cancer antigen [CA 19–9]), and urine analysis. The patient underwent diagnostic ultrasonography using a 12-MHz linear transducer (iU22, Philips Medical Systems, Bothell, Washington). Ultrasonography revealed a 3.7 × 2.9 cm polypoid, mild heterogeneous, and hyperechoic mass in the right upper scrotal wall (Figure 2). The stalk of the mass was connected to the dermis, without extending to the subcutaneous layer. There was no calcification, fat, or cyst in the mass. Color Doppler ultrasonography revealed remarkable intratumoral vascularity in the peripheral portion of the mass from the stalk. The physician determined the best option was surgical excision for diagnosis and treatment of the slowly growing hypervascular tumor; therefore, the patient underwent tumor excision. The tumor was histopathologically diagnosed as EMPD (Figure 3). The tumor invaded the deep dermis without invading the subcutaneous layer. The patient underwent CT, PET/CT, and colonoscopy to exclude underlying malignancies, and these revealed no malignancy or metastasis.

## Discussion

Masses in the scrotal wall are one of the common reasons for patient visits to the urology outpatient department. Skin erosion with mass formation on the scrotal wall is rare, and is suspected to be a malignant tumor.^7–9^ Basal cell carcinoma, squamous cell carcinoma, and cutaneous metastasis from other malignant tumors have been reported as possible causes.^7–9^ EMPD occurs rarely, and is mainly observed in elderly patients.^10^ Because these skin tumors are easily subjected to biopsy, clinicians usually do not perform imaging tests for diagnosis. However, attempts have been made to differentiate skin tumors and delineate margins by using ultrasonography.^11^ Recently, ultrasonography has been principally used to diagnose mass in the scrotal wall and to evaluate the depth of skin invasion.

However, only a few case reports and reviews of the radiologic results of EMPD have been documented,^4–6,12^ and the MRI findings of EMPD in the external genitalia have thus far been reported in only four cases (2 male and two female patients) in two studies.^4,6^ EMPD shows low-to-intermediate signal intensity on T2- and *T*_1_-weighted images, high intensity on diffusion-weighted images, and marked homogeneous enhancement on gadolinium-enhanced *T*_1_-weighted images. Moreover, the vertical invasion of EMPD measured on MRI showed a good correlation with that of the postoperative histopathological results.^4,6^ Ultrasonography is widely available, safe, and relatively inexpensive, and high-resolution ultrasonography can be very useful in evaluating the depth of invasion of the scrotal wall.^5,12^ In a previous report,^5^ high-resolution ultrasonography showed an irregularly contoured and heterogeneous echogenicity mass invading the dermis and subcutaneous layer of the scrotal wall. Furthermore, Doppler ultrasonography revealed increased intralesional vascularity. These ultrasonographic findings are very similar to those observed in our case. Ultrasonography helped predict the extent and vertical invasion of EMPD from the subcutaneous layer to the dermis.

Surgical local wide excision is the treatment of choice for EMPD.^13^ The presence of dermal invasion is the risk factor in distant metastasis and is known to result in a worse prognosis.^14^ Therefore, preoperative information about the depth of EMPD is important. In addition, possible underlying malignancy has been described in EMPD. Prior studies have described gastrointestinal/genitourinary malignancies associated with EMPD.^14–16^ If the EMPD is suspected, extensive examinations (*i.e.* colonoscopy, PET/CT, abdominal CT) are recommended to rule out underlying malignancies. In our patient, despite the use of several diagnostic modalities, no malignancy was detected. The disease is known to have a high frequency of recurrence.^15^ After the 7-month follow-up period, our patient was alive with no evidence of local tumor recurrence, when the surgical scar was visually checked and evaluated by urologists and plastic surgeons in outpatient departments.

In our patient, the main symptom was the palpable mass on the skin of the scrotum. Although there was some erosion on the surface of the mass, mass was not a common sign observed in EMPD. Further, no additional skin lesion was observed around the mass, and the patient did not complain of pain or pruritus. Therefore, our case was more difficult to distinguish from other malignancy (*i.e.* basal cell carcinoma, squamous cell carcinoma, cutaneous metastasis). A previous study reported that 6 of the 25 EMPD in the scrotum presented papillary lesions.^10^ These findings suggested that a few cases revealed papillary or mass-forming EMPD when evaluating scrotal lesion.

In conclusion, mass-forming EMPD is rare and may be confused with other scrotal malignancy. Ultrasonography is a primary imaging modality to evaluate the extent and vertical invasion of EMPD.

## Learning points

1. Ultrasonography is a helpful and safe modality for the exact lesion extent in the scrotal wall.
2. Extramammary Paget’s disease is a rare dermatologic malignancy involving the scrotum. Sonography can predict the depth of invasion of mass-forming extramammary Paget’s disease in the scrotal wall. The lesion extent should be precisely evaluated because the presence of dermal invasion is the risk factor in distant metastasis and is known to result in a worse prognosis.

## Consent

Written consent for publication of the case and imaging data was obtained from the patient and his legal guardian.
